# Protease-inhibitors added to saliva in vitro influence the erosion protective effect of enamel pellicles

**DOI:** 10.1038/s41598-023-35334-x

**Published:** 2023-05-27

**Authors:** Tommy Baumann, Samira Helena Niemeyer, Marília Afonso Rabelo Buzalaf, Thiago Saads Carvalho

**Affiliations:** 1 Department of Restorative, Preventive and Pediatric Dentistry, School of Dental Medicine, University of Bern, Freiburgstrasse 7, CH-3010 Bern, Switzerland; 2 Bauru School of Dentistry, University of São Paulo, Al. Octávio Pinheiro Brisolla, 9-75, Bauru, São Paulo 17012-901 Brazil

**Keywords:** Preventive dentistry, Tooth wear, Tooth erosion

## Abstract

In contrast to pellicles formed in vivo, pellicles formed in vitro provide little to no erosion protection for enamel, possibly due to protein degradation from proteases during pellicle formation. With the objective to achieve a more similar effect as observed for in vivo pellicles, the effects of adding protease inhibitors (PI) to saliva in vitro, and/or exchanging saliva repeatedly during pellicle formation were investigated in a cyclic model of pellicle formation and erosion with human enamel specimens. We repeatedly assessed surface microhardness (SMH), measured initial and final surface reflection intensity (SRI), and determined calcium released during erosion. For all the parameters tested, we observed a clear positive effect on erosion protection when adding PI to saliva for pellicle formation: SMH remained harder, SRI remained higher, and less calcium was released. Additionally, exchanging saliva with fresh one during pellicle formation led to a protective effect, but not as strong as the addition of PI. We conclude that adding protease inhibitors to saliva in vitro for pellicle formation leads to an erosion protective effect, which was further increased by repeatedly exchanging the saliva. Whether the pellicle itself more closely resembles in vivo pellicles remains to be investigated.

## Introduction

Erosive tooth wear (ETW) is a chemical–mechanical process that leads to an irreversible loss of dental hard tissue^1,2^. The chemical part of that process, erosion, is mainly caused by extrinsic or intrinsic acids. These acids, if undersaturated with respect to the dental hard tissue, dissolve dental minerals and thereby soften the surface of the tissue, which then becomes more vulnerable to mechanical challenges.

In a natural oral environment, dental hard tissues are covered by a salivary pellicle. This pellicle starts forming immediately upon contact of saliva with dental hard tissues and serves several functions, among them a protection of the underlying hard tissue from erosive challenges^3^. In the case of enamel, this pellicle is called acquired enamel pellicle (AEP). The AEP forms a semipermeable membrane that hinders the contact of erosive substances with the enamel surface, protecting it from erosion. Furthermore, it also limits the diffusion of dissolved minerals out of the enamel. The erosion-protective effect could be observed in vivo/situ as well as in vitro, but the protection of in vivo/situ formed pellicles is higher than that of the in vitro formed ones^4^.

Upon exposure of the enamel surface to saliva, an electron-dense basal layer is formed within 30 s. Then, a more granular layer forms on top of the basal layer^3^. The basal layer contains the pellicle precursor proteins, which are the main acid resistant proteins^5^, suggesting that this layer is mainly responsible for the erosion protective properties of the pellicle. In vitro pellicles are generally thinner than in vivo/situ pellicles, but proteomics analyses have shown that they contain all the precursor proteins^6^ and are therefore likely composed mainly of the basal layer. Generally, proteomic analysis of in vitro formed pellicles is difficult, since the amount of proteins that can be recovered is small^6^, likely due to the absence of salivary flow to constantly replenish the pellicle. A recent study tried to overcome this by exchanging the saliva several times during the in vitro formation of the pellicle and the authors obtained successful proteomic results^7^.

A proteomic analysis comparing in vitro / in situ / in vivo formed AEPs has revealed various differences between them. Differences were found on the qualitative level as well as on a quantitative level for different proteins^6^. These differences will likely also lead to differences in the properties of the differently formed AEPs, including the erosion protection conferred by them. This is a major drawback for in vitro studies using saliva, as in vitro pellicles cannot fully mimic in vivo pellicles and therefore are of limited use when trying to investigate and explain clinical conditions and phenomena.

Saliva contains proteases from different sources, and therefore shows high proteolytic activity. When working with collected saliva, this activity can change the properties of the saliva over time^8^. Proteolytic degradation of proteins in saliva starts already during collection, and further degradation is observed within 30 min after collection^9^. In vivo, the constant supply of fresh proteins from newly formed saliva counteracts this, which also allows the replenishment or exchange of degraded proteins from the pellicle. In vitro, this is not the case, and while the proteolytic activity of the saliva remains, there is no supply of fresh proteins. This could lead to a different, weakened pellicle containing maximal proteolytically degraded proteins, which would explain the reduced protective properties compared to an in vivo pellicle.

Although in vitro studies can never fully mimic the in vivo situation, it is desirable to come as close as possible. In vitro studies are important for basic research for ethical reasons, to test hypotheses and products for their efficacy before testing them in vivo. The aim of the present study was to determine the effect of adding inhibitors and/or exchanging the saliva during pellicle formation on the erosion protective properties of the pellicle of in vitro formed pellicles, to verify whether the properties would more closely match those observed in vivo. The null hypothesis was that the addition of protease inhibitors and/or the exchange of the saliva during pellicle formation do not have an influence on its protective properties.

## Materials and methods

### Ethics

We complied with approved guidelines and regulations of the local ethics committee (Kantonale Ethikkommission: KEK). The teeth and saliva that we used in this study had been pooled. Both pools were categorized as “irreversibly anonymized” by the local ethics committee (Kantonale Ethikkommission: KEK), and no specific approval from the committee was necessary. In accordance with the guidelines and regulations of the KEK, the volunteers were informed about the use of their teeth or their saliva in research and their informed consent was obtained.

### Teeth/specimen preparation

We prepared 75 enamel specimens from a pool of extracted teeth (stored in 2% chloramine T trihydrate solution). The teeth were embedded in acrylic resin and serially ground flat and polished, removing a total of 200 µm of the outermost enamel. The descending grain sizes used were 18.3 µm, 10 µm, 5 µm, and 3 µm, with a final polish with a grain size of 1 µm just prior to the start of the experimental procedure.

### Saliva collection and preparation

Saliva collection was performed in the mornings, with healthy donors from both sexes, aged 20–40 years, contributing saliva. The donors refrained from eating or drinking for 2 h before saliva collection. Salivary flow was stimulated by chewing on paraffin wax for 10 min, and the stimulated whole saliva was collected in chilled vials. The saliva from all donors was pooled and centrifuged for 20 min at 4 °C (4000 g). The supernatant (cleared saliva) was divided into two parts. Since methanol (MeOH) is the solvent used for the protease inhibitors, it had to be included in both parts. So, to one of the parts, MeOH was added at a dilution of 1:100, then it was divided into small aliquots and stored at −80 °C until use. To the other part, protease inhibitors were added. For that, an inhibitor cocktail containing 100 mM of each Phenylmethanesulfonylfluoride (PMSF), N-Ethylmaleimide (NEM), and Phenanthroline in MeOH was prepared. This cocktail was then mixed with saliva at a dilution of 1:100, resulting in final concentrations of 1 mM of each inhibitor in the saliva. This part of the saliva was then also divided into small aliquots and stored at −80 °C until use.

### Experimental design/procedure

The specimens were randomly divided into 5 groups (n = 15) and underwent an initial assessment of the surface microhardness and reflection intensity. All specimens were subjected to 5 cycles of salivary pellicle formation (2 h, 37 °C, no agitation in a humid chamber), followed by an erosive challenge (6 ml, 1 min, 1% citric acid, pH 3.6, 70 rpm, travel path 50 mm). Between the cycles, the specimens were stored in a humid chamber. The citric acid used for erosion was stored for calcium analyses. After each erosive challenge, the surface microhardness was re-measured. The surface reflection intensity was measured again after the final cycle and after removal of the pellicle remnants.

The 5 groups were: control (ctrl, no pellicle), human saliva (HS), saliva that was exchanged every 30 min, (HS_exch), saliva with protease inhibitors (HS + PI), and saliva with protease inhibitors that was exchanged every 30 min (HS + PI_exch). They differed in the pellicle formation step of the cycles. While the ctrl group was stored in a humid chamber for 2 h without saliva, the HS and HS + PI groups were incubated for 2 h with one aliquot of the according saliva. The HS_exch and HS + PI_exch groups were incubated for a total of 2 h with the according saliva, which was exchanged with a fresh aliquot every 30 min.

### Relative surface microhardness (rSMH)

Surface microhardness (SMH) was measured using a microhardness tester (Knoop diamond, 50 g load, 10 s of dwell time; UHL VMHT Microhardness Tester, UHL technische Mikroskopie GmbH & Co. KG, Asslar, Germany). SMH was measured at baseline (SMH_initial_) and after each erosive challenge (SMH_t_). Six indentations at 25 μm distance from each other were made for each measurement, and the average of the six indentations were defined as the SMH at that time-point. The relative SMH (rSMH) at each time-point was calculated using the formula:

$${\text{rSMH}} = \left( {\frac{{{\text{SMH}}_{{\text{t}}} }}{{{\text{SMH}}_{{{\text{initial}}}} }}} \right) \times 100$$

### Relative surface reflection intensity (rSRI)

The surface reflection intensity (SRI) was measured with a custom-built reflectometer^10^. The maximum value of reflection intensity (SRI value) was registered with a specific software. SRI was measured at baseline (SRI_initial_) and after the final experimental cycle (SRI_end_). Additionally, after the SRI_end_ measurement, the specimens were immersed in 3% NaOCl (5 min, 25 °C, 70 rpm, travel path 50 mm) to remove remnants of the salivary pellicle, and SRI measured once again (SRI_final_). The SRI was then transformed to relative SRI (rSRI), calculated according to the formula:

$${\text{rSRI}} = \left( {\frac{{{\text{SRI}}_{{\text{end/final}}} }}{{{\text{SRI}}_{{{\text{initial}}}} }}} \right) \times 100$$

### Calcium release (CaR)

The concentration of calcium in the citric acid after the erosive challenge was analysed using an atomic absorption spectrometer (AAnalyst 400, Perkin Elmer Analytical Instruments, Waltham, MA, USA). Lanthanum nitrate (0.5%, lanthanum nitrate hexahydrate: La[NO_3_]_3_^.^6H_2_O) was added to the citric acid to eliminate the interference of other ions^11^. The concentration was used to determine the amount of calcium released (CaR) by each specimen. CaR was normalized to the surface area of the specimens, which was determined by taking a picture of the surface using a microscope (Leica, M420, equipped with camera DFC495) with 16 × magnification, and then tracing the contour of the exposed surface with the software program IM500.

### Statistics

We performed statistical analyses using the software R 3.5.3. All significance levels were set at α = 0.05. Data of the different parameters assessed were analysed separately. First, Shapiro–Wilk tests were used to analyse the distribution of the data. Since normal distribution was rejected for some groups, we subsequently performed non-parametric tests. Kruskal–Wallis tests were chosen to analyse whether there were differences between groups. In case of a significant result, we performed post-hoc pairwise comparisons by Wilcoxon rank sum tests with Bonferroni corrections for multiple testing.

To compare the progressions of rSMH, we carried out an ANCOVA, followed by Tukey-HSD test.

## Results

### Relative surface microhardness (rSMH)

**Figure 1:**
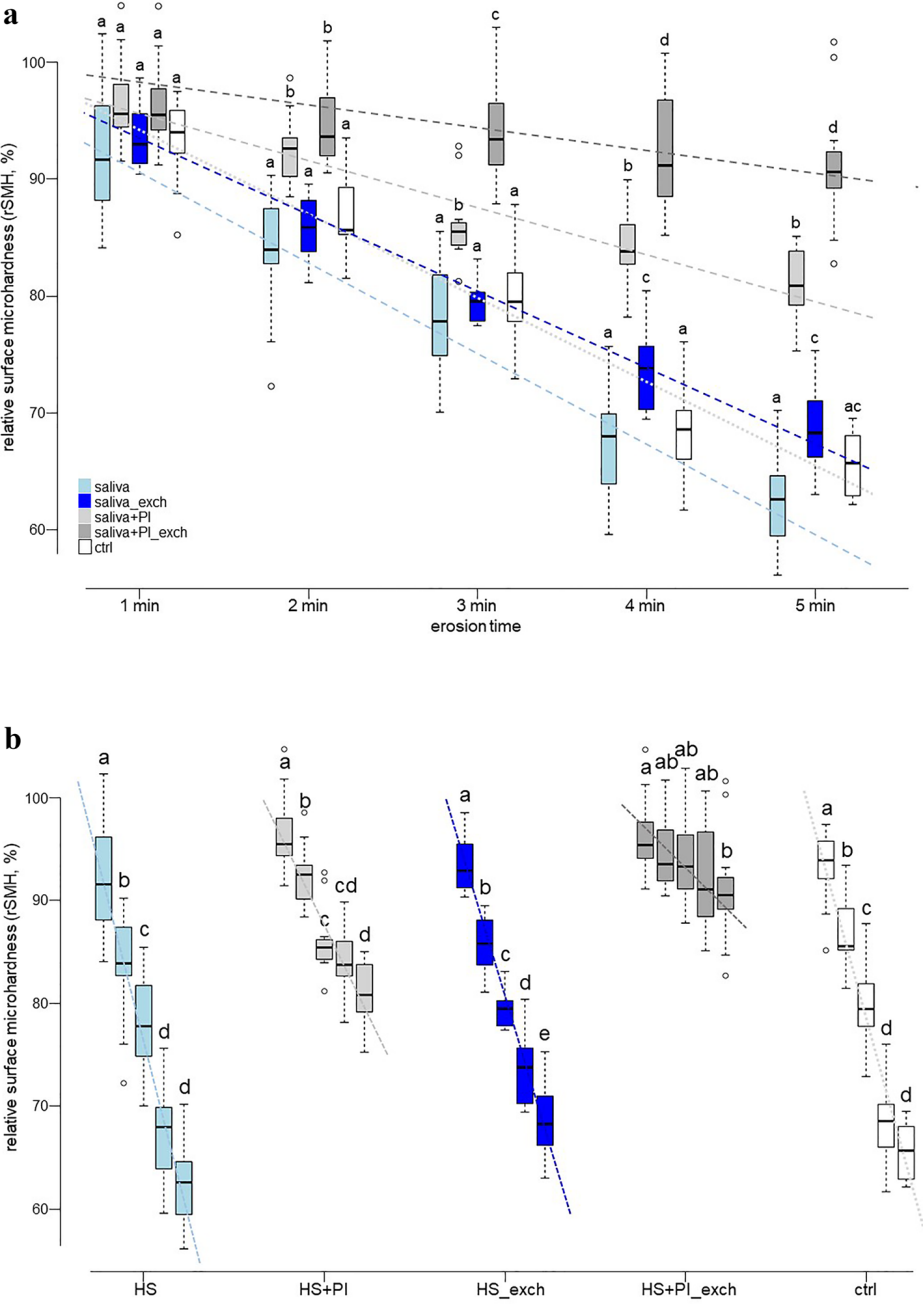
Relative surface microhardness (rSMH) progression of all groups during the course of the five minutes of erosion. (a) rSMH progression over time. Comparisons between the groups were performed separately at each erosion time point. Different letters indicate significant differences between the groups at that specific erosion time point. Dashed lines represent linear fits to the mean rSMH value progression of each group. (b) rSMH progression of the individual groups. Comparisons of the change in rSMH over time were performed separately for each group. Different letters indicate significant differences between the erosion time points within the same group. Dashed lines represent linear fits to the mean rSMH value progression of each group.

After 1 min of erosion, there was not yet a difference between the groups detectable in the relative hardness, but from 2 min on, the groups containing inhibitors in the saliva started to distinct themselves from the other groups (Fig. 1a). At the end of cycling, after a total of 5 min of erosion, the rSMH values (median; IQR) indicated that the group HS + PI_exch (90.5%; 89.2–92.3) best protected enamel against erosion, remaining significantly harder than all the other groups. Although the group HS + PI (80.8%; 79.2–83.8) was softer than the HS + PI_exch group, it was significantly harder than the other groups without inhibitors and the ctrl group. Regarding the groups without inhibitor, the HS_exch (68.2%; 66.2–71.0) group was significantly harder than the HS (62.6%; 59.4–64.6) group, but both of them were not different to the ctrl (65.7%; 62.9–68.0) group.

Analysing the differences between erosion times within each group (Fig. 1b), the ctrl and HS groups showed a pattern where the hardness changed significantly after each erosion within the first 4 min, but did not show differences between the 4th and 5th min anymore. The HS_exch group showed differences between all time-points, but with the overall loss being less than in the former groups. For the groups containing inhibitors, the HS + PI group showed differences within the first 3 min, but then the change slowed and the difference was only significant between the 3rd and 5th min anymore, with the 4th min value in between but not different to any of them. The HS + PI_exch group clearly showed the slowest decrease, with only the 1st and 2nd min being significantly different from the 5th min and all the other in between showing no significant difference (Fig. 1b). Fitting a trendline for the average rSMH values within each group revealed slopes of −1.9 for the HS + PI_exch, −4 for the HS + PI, −6.5 for the HS_exch, −7.2 for the ctrl, and −7.7 for the HS group, indicating that the rates of rSMH decrease differed by a factor of up to four. The groups containing inhibitors lost hardness much slower than the other groups. Analysis of the progression showed that the differences between the groups were significant, except for both HS and HS_exch to ctrl.

### Relative surface reflection intensity (rSRI)

The initial absolute surface reflectivity showed little differences between the groups. There were no significant differences between the groups that received the pellicles, confirming surface uniformity to a certain degree among these groups. The only difference was between the ctrl and the HS + PI groups, where the ctrl group exhibited higher initial absolute reflectivity. Still, to account for small individual differences between the specimens, the reflectivity of each specimen was normalized to its initial value and the resulting relative reflectivity was analysed.

**Figure 2:**
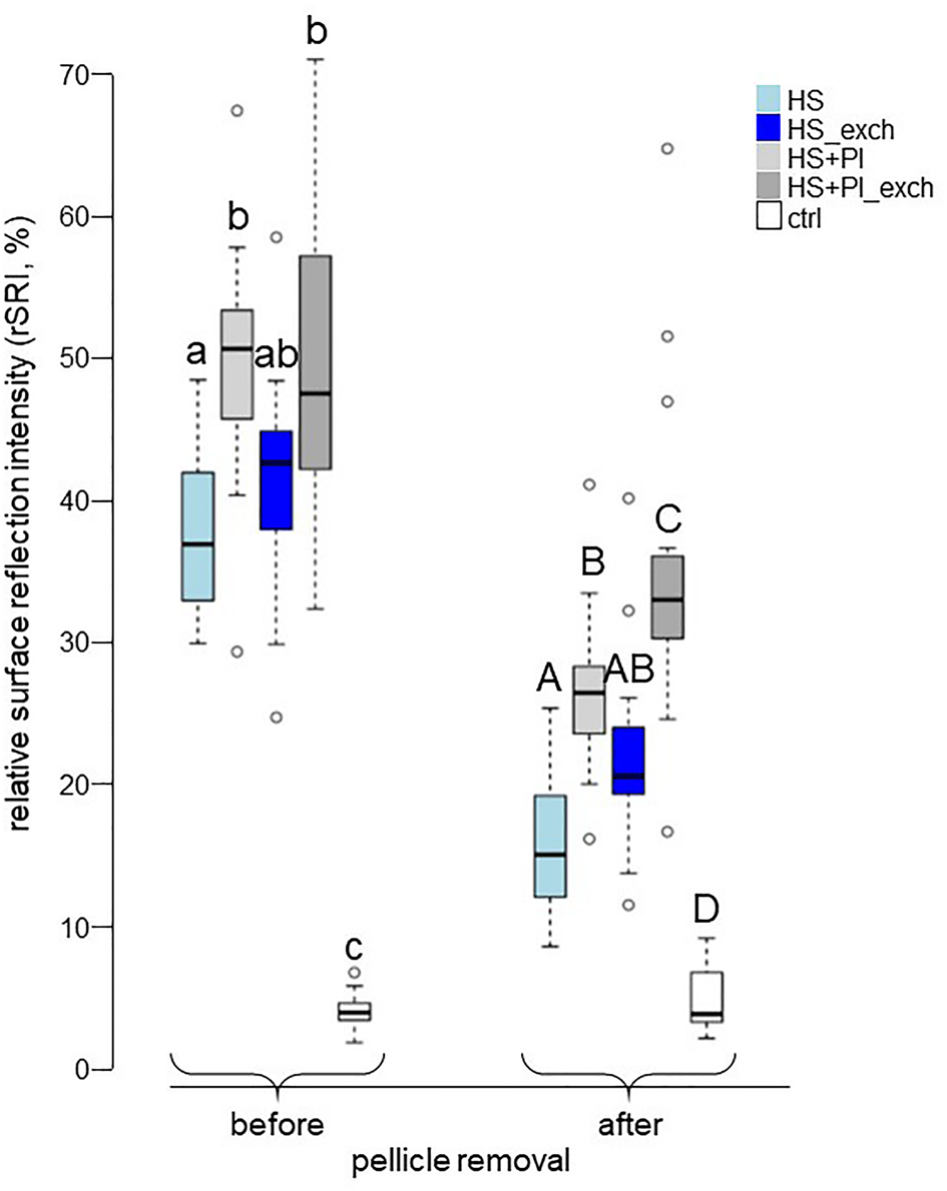
Relative surface reflection intensity (rSRI) at the end of the erosion cycles, before and after pellicle removal. Different lowercase letters indicate significant differences between groups before pellicle removal, different uppercase letters indicate significant differences between groups after pellicle removal.

The remaining relative reflectivity (rSRI) of the surfaces after all cycles is shown in Fig. 2. Before pellicle removal, the ctrl group had significantly lower reflectivity (3.8%; 3.2–4.4) than all other groups. The highest remaining reflectivity was found for the HS + PI group (50.6%; 45.6–53.3). This was followed by the HS + PI_exch (47.4%; 42.1–57.1), the HS_exch (42.5%; 37.8–44.8), and the HS (36.8%; 32.8–41.8) groups. The reflectivity of both groups with inhibitors (HS + PI and HS + PI_exch) did not differ from each other and the HS_exch group, but was significantly higher than the HS group. The groups without inhibitors (HS and HS_excg) did not differ from each other.

After pellicle removal, the ctrl group still had significantly lower reflectivity (3.7%; 3.1–6.6) than all other groups. The significantly highest remaining reflectivity was found for the HS + PI_exch group (32.9%; 30.1–35.9). This was followed by the HS + PI (26.3%; 23.4–28.2), the HS_exch (20.4%; 19.2–23.9), and the HS (14.9%; 11.9–19.1) groups, with the HS + PI group being significantly different to the HS group, but both of them showing no difference to the HS_exch group.

### Calcium release (CaR)

**Figure 3:**
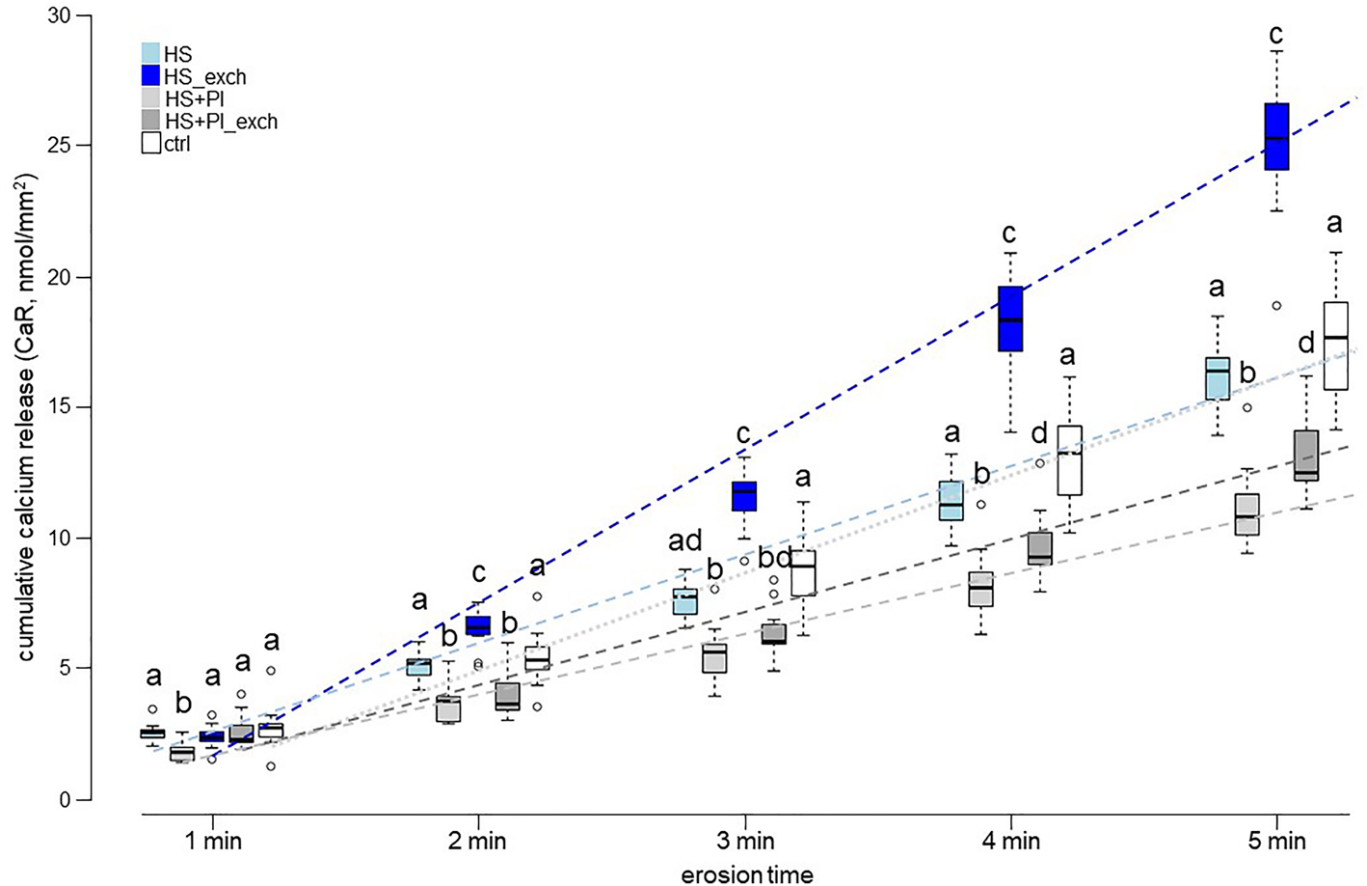
Cumulative calcium release (CaR) of all groups during the course of the five minutes of erosion. Comparisons between the groups were performed separately at each erosion time point. Different letters indicate significant differences between groups at that specific erosion time point. Dashed lines represent linear fits to the mean CaR value progression of each group.

For the cumulative calcium released, the groups containing inhibitors again started to separate from the other groups from 2 min of erosion on with significantly less CaR. The HS + PI group even exhibited less CaR already from the 1st min on. At the end of cycling, the group HS + PI had released the least calcium (10.8 nmol/mm^2^; 10.1–11.7), followed by the group HS + PI_exch (12.58 nmol/mm^2^; 12.3–14.2). The groups HS (16.48 nmol/mm^2^; 15.3–16.9) and ctrl (17.78 nmol/mm^2^; 15.7–19.0) released significantly more calcium than the groups containing inhibitors, with no difference between them. Clearly the most calcium was released by the HS_exch group (25.38 nmol/mm^2^; 24.3–27.4) (Fig. 3).

## Discussion

A positive effect on erosion inhibition by the addition of the inhibitors to saliva in vitro for pellicle formation could be clearly observed in the present study. Therefore, we had to reject our null hypothesis for all the parameters tested. In all three parameters assessed, the groups with inhibitors promoted much better protection than the corresponding groups without inhibitors.

There are no differences in the protective effect of in vitro pellicles with different formation times up to 24 h, with maximum protection reached already after 60 min^4^. Here we used a formation period of 2 h, a commonly used period^12–16^, which should enable us to compare results to earlier studies. As protease inhibitors, we used a mix of chemical, non-peptide inhibitors. Many protease inhibitor cocktails contain peptides as inhibitors, which could interact with, and be integrated into, the pellicle. This would alter the pellicle with peptides that do not naturally occur in the pellicle, which could also lead to an effect on the properties of the pellicle.

We also included a protocol where we exchanged the saliva several times during AEP formation. In vitro pellicles might, compared to in vivo pellicles, suffer from the lack of the possibility to replenish or resupply degraded proteins and peptides, and the rate of saliva replenishment is even a factor that influences inter-individual differences of the pellicle in vivo^17^. Without exchange of saliva, the pellicle is likely thinner and contains fewer proteins, and not enough proteins can be collected from such pellicles for proteomics analyses^7^. For the same type of saliva, our results generally showed a significantly better protection when saliva was exchanged during AEP formation (Figs. 1 and 2). Only the CaR results did not support a better protection by exchanging the saliva, as in those groups more calcium was released than in the corresponding groups without exchange (Fig. 3). One important function of the pellicle is to maintain a high calcium concentration close to the tooth surface to prevent its dissolution^18^. Possibly, the in vitro pellicle is thicker when saliva is exchanged during its formation, so it can also trap more calcium. As calcium-binding proteins will release the bound calcium at low pH^19^, this calcium is then also released while the pellicle disintegrates during erosion, which seemingly heightens the calcium release measured with our protocol^20^. In reality, the calcium released from the enamel surface was most likely less in the groups where saliva was exchanged, since the rSMH decreased significantly less. However, the effect observed when adding the protease inhibitors is much greater than the one observed when exchange of the saliva is included in the protocol.

While our results clearly show an erosion inhibiting effect of in vitro pellicles when protease inhibitors are added to saliva, an explanation for this effect cannot be directly deduced. One possible explanation could be that there are more intact proteins in the pellicle, since the proteolytic activity is inhibited. If this is the case, the differences to in vivo formed pellicles might even be increased, as in vitro formed pellicles already contain more intact salivary proteins than the in vivo formed pellicle^21^. In vivo formed pellicles contain many peptide fragments, and proteolysis of the original proteins can occur before or after their adsorption to tooth minerals^22^. Since binding to hydroxyapatite protects some pellicle proteins from cleavage^23^, but they are found in the pellicle in intact^24^ as well as in a cleaved form^25^, a large part of the processing of the pellicle proteins must happen in saliva before adsorption to the tooth surface. Protease inhibitors would prevent this processing, but one has to keep in mind that a saliva sample used in vitro is a closed system, while the oral environment is an open system with constant resupply of fresh proteins and clearance of processed saliva^26^. In the closed system, there might be some “overprocessing” of the salivary proteins, especially during the 2 h of incubation used for pellicle formation, with no resupply of fresh proteins.

Another effect of the inhibitors could be that the pellicle gets thicker or denser. Proteomics results, where inhibitors are necessary to collect enough material for analysis^7^, would hint to thicker pellicles. Our CaR results, on the other hand, suggest that there are also some qualitative differences. As explained above, thicker pellicles can store more calcium and release it upon erosion, leading to seemingly higher CaR than in the ctrl group, as can be observed for the HS_exch group. However, the results showed that the groups containing inhibitors released significantly less calcium than the ctrl group (Fig. 3). If the effect of the inhibitors was only to enable the pellicle to get thicker, one would expect even larger amounts of calcium to be stored in the pellicle and to be released upon erosion, which would lead to a seemingly much higher calcium release. As the opposite is the case, there also have to be qualitative differences induced by the presence of the inhibitors. A possibility could be that the pellicle is denser and less sensitive to acid attacks. Consequently, it would release less of its stored calcium, while it would also limit diffusion of acids to the surface and of dissolved minerals away from the surface, explaining the good protection observed in all the parameters measured.

The SRI is an indirect measurement of the surface roughness. The pellicle layer causes a certain smoothing of the roughness, leading to higher reflection intensities^27^, which were also observed here before pellicle removal. Only the ctrl group, which did not receive a pellicle, still had similar rSRI values after pellicle removal (Fig. 2). The real differences between groups in surface roughness can be seen after pellicle removal. These differences were rather similar to the differences with the pellicle still present. This would mean that all the pellicles had a similar smoothing effect to the surface, and the differences visible before pellicle removal already mainly stem from differences in surface roughness. Therefore, we assume that the (ultra-) structure of the different pellicles was rather similar.

Classical analysis methods to assess enamel demineralization were chosen for the present study, namely hardness, calcium release^11^, and surface reflection, which is an indirect measurement of roughness. One often used method to measure surface loss by erosive tooth wear, profilometry, was not performed here, since the short erosion times used here, to simulate early erosion, lead to very limited surface loss with high variation between specimens. Other methods, such as AFM, are perfectly suited to investigate surface properties and structure of both the substrate and the pellicle, which help to understand the mechanism of protection and the interaction between pellicle and substrate, but less so the magnitude of the protective effect and demineralization, which was the focus of the present study. Furthermore, electron microscopy techniques or neutron reflectometry would be helpful to characterize the ultrastructure of the pellicles^11,28^. Having observed a dramatic effect by the inhibition of the proteases, these methods will be helpful to investigate the underlying reasons for this effect.

Although the overall goal is to get closer to an in vivo or in situ formed pellicle, it is not possible to say whether the pellicles formed here more closely resembled in vivo pellicles. Direct functional comparisons would be necessary between in vitro pellicles with inhibitors and in situ formed pellicles to shed more light on how closely they resemble each other. A shortcoming of the present study is, hence, that an in situ pellicle group was not included in the study protocol. This will be important for future studies, together with investigations of the pellicles themselves, analysing e.g. the ultrastructure by electron microscopy techniques. Although this would provide mainly qualitative information, it might help visualize and explain the differences between the pellicles and their protection of the enamel surface. Furthermore, quantitative differences could be analysed by proteomic analyses. Although in vitro pellicles have already been compared to in situ and in vivo pellicles by proteomics methods, these pellicles were already formed with protease inhibitors present^6^, and there is no comparison to in vitro pellicles formed in the classical way without inhibitors. Concerning the saliva used for pellicle formation, not only the proteases have an effect on its properties, but also collection and processing^8^. Investigating different collection and processing protocols might also help identifying ways to achieve in vitro pellicles that resemble in vivo pellicles more closely.

The present study showed dramatic differences between the protective properties of in vitro pellicles formed with saliva either with or without added protease inhibitors. This might be an important step toward developing in vitro models for saliva research that more closely resemble in vivo conditions, since some basic research is nearly impossible to carry out in vivo.

## Data Availability

The datasets generated during and/or analysed during the current study are available from the corresponding author on reasonable request.

## References

[1] Carvalho TS (2015). Consensus report of the European Federation of Conservative Dentistry: Erosive tooth wear–diagnosis and management. Clin. Oral Invest..

[2] Schlueter N (2020). Terminology of erosive tooth wear: Consensus report of a workshop organized by the ORCA and the cariology research group of the IADR. Caries Res..

[3] Hannig M, Hannig C (2014). The pellicle and erosion. Monogr. Oral Sci..

[4] Hannig M (2002). The protective nature of the salivary pellicle. Int. Dent. J..

[5] Delecrode TR (2015). Identification of acid-resistant proteins in acquired enamel pellicle. J. Dent..

[6] Pelá VT (2020). Proteomic profiles of the acquired enamel pellicle formed in vitro, in situ, or in vivo. Eur. J. Oral Sci..

[7] Pelá VT, Ventura TMO, Buzalaf MAR (2020). Optimizing the formation of the acquired enamel pellicle in vitro for proteomic analysis. J. Appl. Oral Sci. Revista FOB.

[8] Schipper RG, Silletti E, Vingerhoeds MH (2007). Saliva as research material: biochemical, physicochemical and practical aspects. Arch. Oral Biol..

[9] Esser D (2008). Sample stability and protein composition of saliva: Implications for its use as a diagnostic fluid. Biomark. insights.

[10] Rakhmatullina E (2011). Application of the specular and diffuse reflection analysis for in vitro diagnostics of dental erosion: Correlation with enamel softening, roughness, and calcium release. J. Biomed. Opt..

[11] Schlueter N, Hara A, Shellis RP, Ganss C (2011). Methods for the measurement and characterization of erosion in enamel and dentine. Caries Res..

[12] Carvalho TS, Pham KN, Niemeyer SH, Baumann T (2021). The effect of red wine in modifying the salivary pellicle and modulating dental erosion kinetics. Eur. J. Oral Sci..

[13] Sieber KR, Schmidt C, Baumann T, Lussi A, Carvalho TS (2019). Acquired enamel pellicle modification with casein and mucin in different concentrations and its impact on initial dental erosion. Caries Res..

[14] Rakhmatullina E, Beyeler B, Lussi A (2013). Inhibition of enamel erosion by stannous and fluoride containing rinsing solutions. Schweizer Monatsschrift fur Zahnmedizin = Revue mensuelle suisse d'odonto-stomatologie = Rivista mensile svizzera di odontologia e stomatologia/SSO.

[15] Hove LH, Holme B, Young A, Tveit AB (2007). The erosion-inhibiting effect of TiF4, SnF2, and NaF solutions on pellicle-covered enamel in vitro. Acta Odontol. Scand..

[16] Carvalho TS, Baumann T, Lussi A (2016). In vitro salivary pellicles from adults and children have different protective effects against erosion. Clin. Oral Invest..

[17] Mutahar M (2017). Reduced statherin in acquired enamel pellicle on eroded teeth compared to healthy teeth in the same subjects: An in-vivo study. PloS one.

[18] Hay, D. I. & Bowen, W. H. in *Saliva and Oral Health* (eds W. M. Edgar & D. M. O'Mullane) 105–122 (Thanet Press, Margate, 1996).

[19] Carpenter G (2014). Composition of enamel pellicle from dental erosion patients. Caries Res..

[20] Baumann T, Bereiter R, Lussi A, Carvalho TS (2017). The effect of different salivary calcium concentrations on the erosion protection conferred by the salivary pellicle. Sci. Rep..

[21] Yao Y (2001). Compositional analysis of human acquired enamel pellicle by mass spectrometry. Arch. Oral Biol..

[22] Siqueira WL, Custodio W, McDonald EE (2012). New insights into the composition and functions of the acquired enamel pellicle. J. Dent. Res..

[23] McDonald EE, Goldberg HA, Tabbara N, Mendes FM, Siqueira WL (2011). Histatin 1 resists proteolytic degradation when adsorbed to hydroxyapatite. J. Dent. Res..

[24] Siqueira WL, Margolis HC, Helmerhorst EJ, Mendes FM, Oppenheim FG (2010). Evidence of intact histatins in the in vivo acquired enamel pellicle. J. Dent. Res..

[25] Siqueira WL, Oppenheim FG (2009). Small molecular weight proteins/peptides present in the in vivo formed human acquired enamel pellicle. Arch. Oral Biol..

[26] Hannig M, Joiner A (2006). The structure, function and properties of the acquired pellicle. Monogr. Oral Sci..

[27] Lussi A (2012). Effects of enamel abrasion, salivary pellicle, and measurement angle on the optical assessment of dental erosion. J. Biomed. Opt..

[28] Gonzalez-Martinez JF (2022). MUC5B mucin films under mechanical confinement: A combined neutron reflectometry and atomic force microscopy study. J. Colloid Interface Sci..

